# Derivation and Validation of an Algorithmic Classification of Early Cognitive Impairment

**DOI:** 10.1093/geroni/igab046.1696

**Published:** 2021-12-17

**Authors:** Alden Gross, Yang An, Frank Lin, Luigi Ferrucci, Jennifer Schrack, Yuri Agrawal, Susan Resnick

**Affiliations:** 1 Johns Hopkins Bloomberg School of Public Health, Baltimore, Maryland, United States; 2 NIA, Baltimore, Maryland, United States; 3 Johns Hopkins University, Johns Hopkins University, Maryland, United States; 4 National Institute on Aging, Baltimore, Maryland, United States; 5 Otolaryngology, Baltimore, Maryland, United States

## Abstract

The long prodromal period for dementia pathology demands valid and reliable approaches to detect cases before clinically recognizable symptoms emerge, by which time it may be too late to effectively intervene. We derived and compared several algorithms for early cognitive impairment (ECI) using longitudinal data on 1704 BLSA participants. Algorithms were based on cognitive impairment in various combinations of memory and non-memory tests, and the CDR. The best-performing algorithm was defined based on 1SD below age-and race-specific means in Card Rotations or California Verbal Learning Test immediate recall, two tests that in prior work show the earliest declines prior to dementia onset. While this ECI algorithm showed low concordance with concurrent adjudicated MCI/dementia (AUC: 0.63, sensitivity: 0.54, specificity: 0.73), it was among the best predictors of progression to MCI/dementia (HR: 3.65, 95% CI: 1.69,7.87). This algorithm may be useful in epidemiologic work to evaluate risk factors for early cognitive impairment.

